# Sequential Exercise in Triathletes: Variations in GH and Water Loss

**DOI:** 10.1371/journal.pone.0096145

**Published:** 2014-04-24

**Authors:** Olivier Galy, Karim Chamari, Christelle Peyreigne, Jacques Mercier, Olivier Hue

**Affiliations:** 1 Laboratory ACTES, (EA 3596: Department of Physiology), Faculty of Physical Activity and Sports, University of Guadeloupe, Pointe à Pitre, France; 2 Laboratory CNEP (EA 4242), University of New Caledonia, Nouméa, New Caledonia; 3 Athlete Health and Performance Research Centre, Aspetar, Qatar Orthopaedic and Sports Medicine Hospital, Doha, Qatar; 4 Laboratory INSERM (U 1046), University of Montpellier 1 and 2, Montpellier Hospital, France; 5 Faculty of Physical Activity and Sports (EA 2991), University of Montpellier 1, Montpellier, France; Universidad Europea de Madrid, Spain

## Abstract

Growth hormone (GH) may stimulate water loss during exercise by activating sweating. This study investigated GH secretion and water loss during sequential cycling and running, taking postural changes into account. The two exercise segments had similar durations and were performed at the same relative intensity to determine their respective contributions to water loss and the plasma volume variation noted in such trials. Eight elite triathletes first performed an incremental cycle test to assess maximal oxygen consumption. Then, the triathletes performed one of two trials in randomized order: constant submaximal cycling followed by treadmill running (C_1_-R_2_) or an inversed succession of running followed by cycling (R_1_-C_2_). Each segment of both trials was performed for 20 minutes at ∼75% of maximal oxygen consumption. The second trial, reversing the segment order of the first trial, took place two weeks later. During cycling, the triathletes used their own bicycles equipped with a profiled handlebar. Blood sampling (for GH concentrations, plasma viscosity and plasma volume variation) was conducted at rest and after each segment while water loss was estimated from the post- and pre-measures. GH increases were significantly lower in R_2_ than C_2_ (72.2±50.1 vs. 164.0±157 ng.ml^−1^.min^−1^, respectively; *P*<0.05). Water loss was significantly lower after C_1_-R_2_ than R_1_-C_2_ (1105±163 and 1235±153 ml, respectively; P<0.05). Plasma volume variation was significantly negative in C_1_ and R_1_ (−6.15±2.0 and −3.16±5.0%, respectively; *P*<0.05), not significant in C_2_, and significantly positive for seven subjects in R_2_ (4.05±3.1%). We concluded that the lower GH increases in R_2_ may have contributed to the smaller reduction in plasma volume by reducing sweating. Moreover, this lower GH response could be explained by the postural change during the transition from cycling to running. We recommend to pay particular attention to their hydration status during R_1_ which could limit a potential dehydration during C_2_.

## Introduction

It is now widely acknowledged that growth hormone (GH) secretion is strongly stimulated by exercise [1]. Several studies have further suggested that GH facilitates water loss by stimulating sweating [2], [3], [4], [5], [6], [7], [8]. For example, Juul et al. [2] observed that subjects with a GH deficiency sweated less during exercise than healthy subjects. In highly trained athletes, our group showed that the water loss recorded during exercise was positively correlated with the exercise-induced GH response [9]. The direct impact of GH on sweating has been supported by histological research demonstrating specific GH receptors localized on the epithelium of sweat glands [10]. Moreover, our group also showed that hypohydration reduced the exercise-induced GH response, leading to the hypothesis that the GH response itself is under the control of baroreceptors [8]. While GH response has been studied during many types of exercise, the combined effects of GH response and sweating in multi-sport activities like the triathlon or duathlon and the order of activities (i.e., cycling and running) could be implicated in performance. We suspect that such trials would induce a specific GH response and water loss. Indeed, triathletes assume a characteristic position on their bikes, with the trunk maintained at a less than 30° head-up tilt from the horizontal. During the brief transition to running, a rapid passage from this position to the upright position occurs, and it has been shown that the rapid transition of the trunk from horizontal to vertical head-up posture or upright posture stimulates the baroreceptor reflex, leading to increased heart rate [11], [12]. It is also well recognized that the hormonal regulation induced by baroreceptor activation leads to water retention [8] and, indeed, an expanded plasma volume during running subsequent to cycling [13], [14]. Previous work [14] did not investigate hormonal responses and excluded the role of sweating in the observed phenomenon. However, the experiment was performed in the actual conditions of a short-distance triathlon, with different durations of the cycling and running segments and without measurement of the relative intensity during the segments; in addition, the triathletes were allowed to drink ad libitum. In a later study, our group did not measure hormonal responses either but found similar plasma volume expansion during isolated run and cycle segments [15] and a run subsequent to cycling during controlled trials [13]. So, during the transition phase between C_1_ and R_2_, as well as during subsequent run of C_1_-R_2_, the combinated effects of baroreceptor reflex, HR increases could be together implicated in the GH response and accentuate dehydration.

The aim of the present study was therefore 1) to investigate GH secretion and water loss in laboratory during cycling of the running-cycling sequence (C_2_ of R_1_-C_2_) and running of the cycling-running sequence (R_2_ of C_1_-R_2_) of similar relative intensity and duration, and 2) to determine whether these parameters contribute to the expanded plasma volume during sequential running, as previously described.

## Materials and Methods

### Subjects

**Figure 1:**
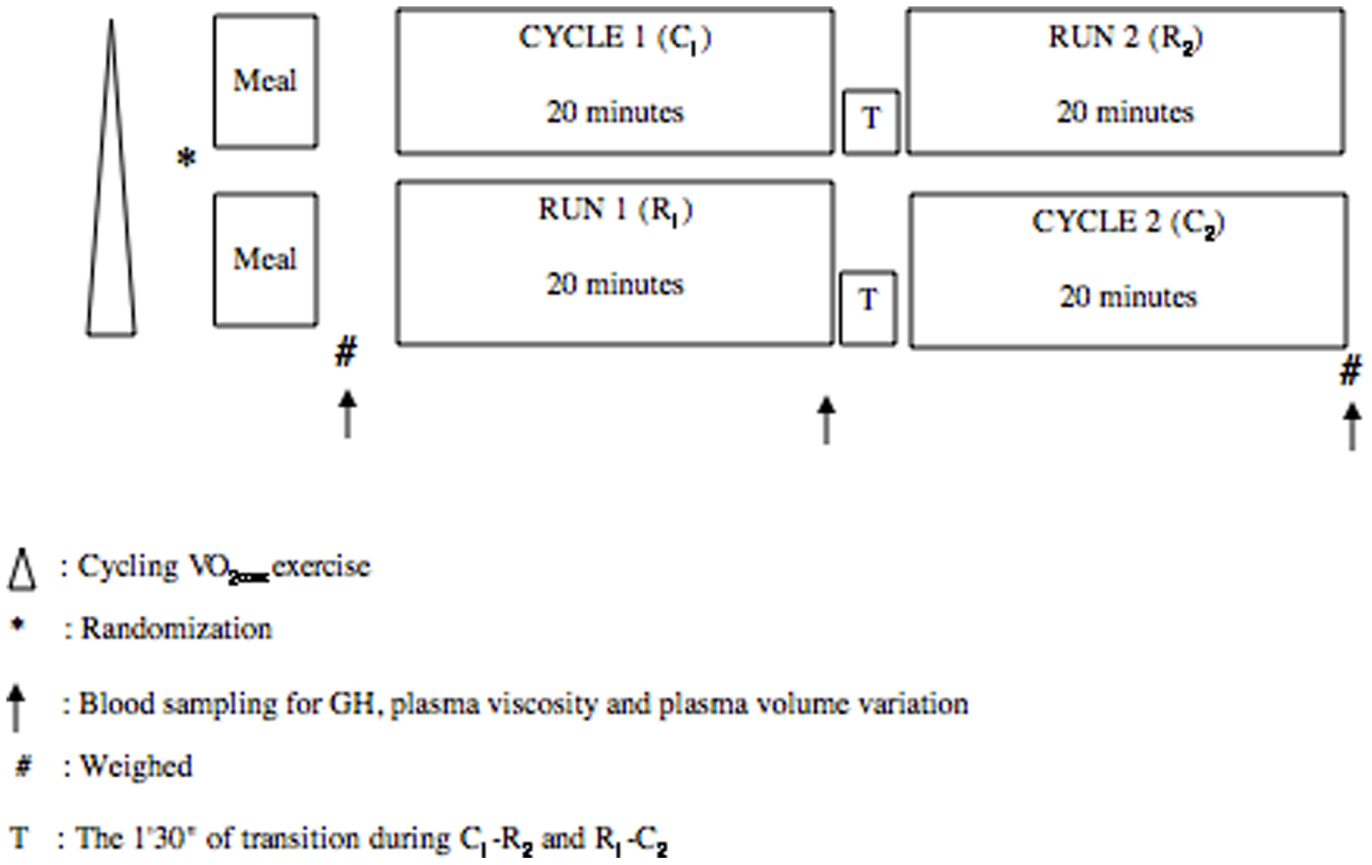
The experimental protocol. Maximal cycling exercise as the first step. One week later, after randomization and a standardized breakfast two hours before the trials, the triathletes performed the cycle-run (C_1_-R_2_) or run-cycle successions (R_1_-C_2_) and, two weeks days later, they performed the second succession in similar conditions.

Eight elite male triathletes participated in this study (Figure 1). All were students and members of the university athletic team, which has been national university triathlon champion for five consecutive years. The triathletes also belonged to the French federation team. They regularly competed in the European Triathlon Union Cup, the International Triathlon Union Cup, and short-distance national triathlon championships. All athletes had competed at the elite level for four to ten years preceding the study. In these conditions, we assume that, although the small size of the group might constitute a methodological limitation, it is nevertheless compensated by the high level and expertise of these triathletes. All trials were performed at the start of the competitive season; for further details on a typical season, please see Galy et al. [16]. The triathletes were 22.1±2.9 years old, with a weight of 69.3±6.4 kg and a height of 181.6±7.4 cm (Table 1). They were non-smokers without histories of pulmonary or cardiovascular disease. Training included swimming, cycling, and running for 5.6±1.6, 9.5±2.4, and 2.7±0.9 hours.week^−1^, respectively. None of the athletes were taking medication or had been injured in the three months preceding the study. They were familiarized with treadmill and cycle ergometer use in the first session and they performed maximal exercise in the second session. They gave written consent to participate in this study after the purpose, design and risks had been described. The study protocol complied with legal requirements and the declarations of Helsinki and was approved by the University Ethics Committee of the University of Montpellier I, Montpellier, France.

**Table 1:** Anthropometric characteristics, cardiorespiratory performances during maximal cycle ergometry, and training characteristics in all subjects.

	Age (years)	Body mass (kg)	Height (cm)	Maximal oxygen consumption (ml.min^−1^.kg^−1^)	Years of competition (years)	Training volume (hours.weeks^−1^)
**Mean (SD)**	22.1 (±2.9)	69.3 (±6.4)	181.6 (±7.4)	69.4 (±3.3)	6.6 (±2.6)	18.3 (±4.2)

Means are expressed in SD.

### Testing Protocol

Testing protocol is presented in Figure 1. Two hours before the experiment, a standard meal was given to the triathletes, as in a previous study from our group [8]. The subjects ate a standardized breakfast containing 2070 kJ and comprising 9.1% proteins, 27.5% lipids and 63.4% carbohydrates. The meal was composed of bread (80 g), butter (10 g), jam (20 g), milk (80 ml), sugar (10 g) and powdered coffee (2.5 g) or tea, with no additional water supplementation. This meal was shown to restore normal glycemia in less than two hours [17]. An incremental cycle test was first performed in the morning on a mechanical cycle ergometer (Monark 864, Monark-Crescent AB, Varburg, Sweden) to assess maximal oxygen consumption. Ventilatory data were measured every minute using mass spectrometry with an automated breath-by-breath system (MGA-1100, Marquette, NY). Three to seven days later, the triathletes performed one of two trials in randomized order: constant submaximal cycling followed by treadmill running (C_1_-R_2_) or an inversed succession of running followed by cycling (R_1_-C_2_). Each segment of both trials was performed for 20 minutes at ∼75% of maximal oxygen consumption. The second trial, reversing the segment order of the first trial, took place two weeks later at the same time of day in the morning and on the same day of the week to minimize the effects of circadian rhythms and personal training. The incremental test and the trials were conducted in an air-conditioned laboratory with a mean room temperature of 21.9±0.2°C and a barometric pressure of 777.5±4.5 mm.Hg and 50% of humidity. The triathletes were asked to maintain their training schedule for the duration of the study but they were not allowed to compete in a triathlon during the testing period. The triathletes were also asked to refrain from training on experimental days. The protocol of the incremental cycle test started with a 3-min warm-up at 30 W. The power was then increased by 30 W every minute until each subject reached volitional fatigue. Because our group had previously demonstrated that maximal oxygen consumption during incremental cycling and running were similar in young triathletes without prior athletic specialization [18] –which was the case of the triathletes of the present study–the cycling and running activities were managed to maintain VO_2_ close to that of the ventilatory threshold measured in trial 1 and to simulate competitive conditions (∼ 75% of VO_2max_). During cycling, the triathletes used their own bicycles on a home trainer (Pro Training, Pro Trainer, Milan, Italy). Each cycle was equipped with a profiled handlebar commonly used during the cycle segment of a triathlon. The athletes assumed the typical triathlon posture, with the trunk maintained at a less than 30° head-up tilt from horizontal. The triathletes reached cycling speed in less than 1 min and the VO_2_ level at approximately the 3^rd^ minute. It was then maintained by increasing or decreasing the cycling speed by 1 km.h^−1^ every minute. Distances were recorded with a bike odometer (Top Bike, Tokyo, Japan). After C_1_ a short active transition of ∼1 min 30 s allowed the triathletes to provide blood samples, change their shoes and then get on the treadmill (Gymroll 1800, Gymroll, Roche la Molière, France). During R_2_, the triathletes reached running speed in less than 1 min and the VO_2_ level at approximately the 3^rd^ minute. It was then maintained by increasing or decreasing the running speed by 0.5 km.h^−1^ every minute. Distances were recorded with the treadmill odometer. During the opposite trial, after R_1_, a short active transition of ∼1 min 30 s allowed the triathletes to provide blood samples, change their shoes, and then get on their bicycles for C_2_ in similar conditions than in the opposite trial. In each trial segment, heart rate (HR: beats.min^−1^) was measured with a telemetry system (Polar Sport Tester, Polar Electro Oy, Kempele, Finland).

### Water Loss

The water loss was estimated by the difference in naked weight measured just before and immediately after the end of the complete trial on a precision scale (Model F 150-S-F2, precision 1 g; Sartorius, Goettingen, Germany). We asked the triathletes to urinate before weight measurements were taken. For the 15 min preceding the onset of trials, they remained on the cyclo-ergometer. The athletes were asked to wear the same triathletes’ cycling clothing and shoes for both sessions. They wore cycling shorts but no tops on both occasions.

### Blood Sampling

A venous catheter was inserted into a superficial forearm vein by the medical staff before the sequential trials to allow sampling for measurement of GH concentrations, plasma viscosity and plasma volume variation. A three-way tap was placed on the catheter to allow rinsing with a syringe containing a mixture of heparin and physiological saline (250 IU.ml^−1^) and blood sampling with a dry syringe after the catheter had been cleared of saline. Approximately 5 ml of blood was sampled at rest and during the last 20 s of segment 1 (R_1_ or C_1_) and during the last 20 s of segment 2 (C_2_ or R_2_) of each sequential trial. At rest, blood sampling was done in the position of the segment the triathlete was about to start (i.e., running or cycling using the handlebar).

### Plasma Viscosity

Blood samples for plasma viscosity measurements were drawn into Vacutainer tubes (Becton, Dickinson and Company, Franklin Lakes, NJ), with potassium EDTA as the anticoagulant. They were centrifuged for 15 min at 3000 rpm. One milliliter of plasma was collected in a specific syringe and plasma viscosity was measured at a high shear rate (mPa.s) with a falling ball viscometer (MT 90 Medicatest, Saint Benoit, France) [19]. The coefficient of variation for this method ranged between 0.6 and 0.8% [20].

### Plasma Volume Variation

Changes in plasma volume (%ΔPV) during exercise were assessed from hematocrit changes with a formula previously used during moderate and maximal exercise [21], [22], [23]. This formula does not require hemoglobin measurements and produces values in close agreement with the measured values [21], [24]. The formula can be reduced to: %ΔPV = [10000×(Ho-H)]/[Ho×(100-Ho)] where Ho is resting hematocrit and H is hematocrit during exercise. The percentage of total plasma variation at the end of each segment 1 was calculated from hematocrit at rest and the end of segment 1. The percentage of total plasma variation at the end of each segment 2 was calculated from hematocrit at the end of segment 1 and the end of segment 2. Hematocrit was determined in duplicate with a small amount of blood collected from each blood sample in a microcapillary tube. This latter underwent microcentrifugation for 5 minutes at 15 g. Values were obtained using a specific standard rule. The coefficient of variation of the method was about 0.9% [9].

### GH Assay

GH was assayed by immunoradiometry with the ELSA-hGH kit (CIS Bio International, Gif-sur-Yvette, France) using two monoclonal mouse antibodies. The intra- and inter-assay coefficients of variation were 2.5% and 3.8%, respectively, with a sensitivity of 0.1 ng.ml^−1^. Since some of the apparent GH increase during exercise results from hemoconcentration, we corrected values after each segment of C_1_-R_2_ and R_1_-C_2_ for plasma volume contraction. For example corrected GH after C_1_ = GH at C_1_ − ((GH at C_1_×%ΔPV of C_1_)/100)_._ these values reflected corrections GH increases effectively observed during exercise without dilution effects.

### Statistical Analysis

The results are expressed as means ± SD. Distances performed during cycling (C_1_ or C_2_) and running (R_1_ or R_2_) were compared using a paired Student *t*-test. After verification of a normal distribution (Gaussian graphical distribution) using the Shapiro-Wilk W-test, µb, µp, ΔPV and the cardioventilatory data were recorded throughout trials 1 and 2. The values of VO_2_ and HR were compared using a 2**-**way analysis (exercise × time) of variance (ANOVA) with repeated measures.

GH, µb, µp and ΔPV measurements of trials 1 and 2 were compared to determine the influence of specific exercise on these parameters using a 1-way ANOVA. The GH area under the curve (GH AUC) was calculated with the trapezoidal rule and values were compared with a paired t-test. The relationship between GH increase and plasma volume variation was assumed by Pearson correlation and was calculated using the SYSTAT program. Statistical significance was accepted at the *P*<0.05 level.

## Results

No significant difference was observed between the distances covered in C_1_ and C_2_ or between the distances covered in R_1_ and R_2_ (Table 2). The maximal oxygen consumption values are reported with subject characteristics in Table 1. The oxygen consumption level and the relative intensity are reported in Table 2; no statistical difference was observed between the segments of each trial for these values. During each trial, significant differences were observed between GH concentrations at rest and at the end of segment 1 (R_1_ or C_1_), between those at rest and at the end of segment 2 (C_2_ or R_2_), and between those at the end of segment 1 and the end of segment 2 (*P*<0.05) (one subject value was missing for C_2;_ Table 3).

**Table 2:** Distances covered; absolute and relative intensity of each trial.

	R_1_	T_ransi_	C_2_	C_1_	T_ransi_	R_2_
**Distances** (km)	6.6 (±0.01)		12.4 (±1.2)	12.3 (±1.1)		6.8 (±0.1)
**Oxygen consumption level** (ml.min^−1^.kg^−1^ **)**	52.1 (±1.2)		51.1 (±2.3)	52.1 (±3.2)		51.3 (±1.5)
**Relative intensity** (% of maximal oxygen consumption)	74.0 (±5.0)		75.3 (±5.2)	75.4 (±4.5)		73.5 (±3.6)

Means are expressed in SD. T_ransi_: active short transition 1 min 30 s.

**Table 3:** GH, plasma volume variation and plasma viscosity during trials.

	Rest	R_1_	T_ransi_	C_2_	Rest	C_1_	T_ransi_	R_2_
**GH concentration** [ng ml^−1^]	0.65 (±0.8)	5.70 ^a^ (±3.1)		20.50 ^a,b^ (±2.7)	3.10 (±4.1)	10.10^ a^ (±10.0)		16.85^ a,b^ (±6.1)
**GH increase** [ng ml^−1^.min^−1^]	–	50.5 (±40.7)		164.0 ^b,c^ (±15.7)	–	60.0 (±45.5)		72.2 ^c^ (±50.1)
**Plasma volume variation** (%)	–	−3.16 ^a^ (±5.0)		−2.0 ^c^ (±3.2)	–	−6.15 ^a^ (±2.0)		4.05 ^b,c^ (±3.1)
**Plasma viscosity** (mPa s)	1.43 (±0.04)	1.47^a^ (±0.06)		1.47^a^ (±0.05)	1.44 (±0.08)	1.51^a^ (±0.1)		1.47^a,b^ (±0.05)

Means are expressed in SD. T_ransi_, brief active transition of 1 min 30 s.

a: significantly different from rest, *P*<0.05.

b: significantly different from the precedent exercise, *P*<0.05.

c: significantly different from the second segment of the opposite trial, *P*<0.05.

No significant differences were observed between the resting GH values of the two trials or between the values obtained at post-segments 1 or post-segments 2. The GH-AUC in segment 2 was significantly lower for running than for cycling (*P*<0.05), whereas no significant difference was noted for segment 1 (Table 3). A significant negative variation in plasma volume was observed in segment 1 of both trials (*P*<0.05), no significant variation was observed in C_2_, and a significant positive variation was observed in R_2_ (*P*<0.05) (Table 3).

**Figure 2:**
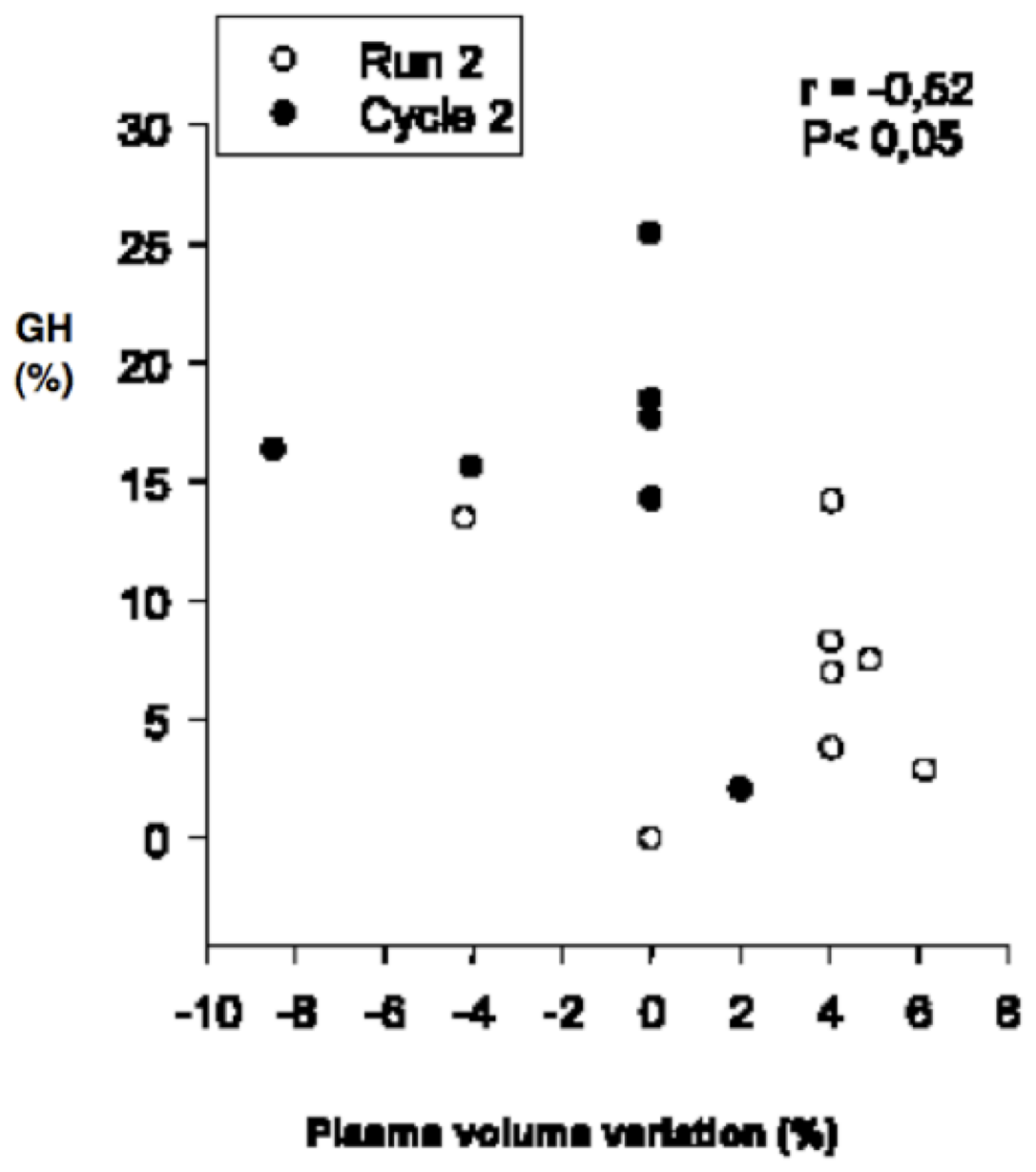
Spearman correlation between the plasma volume variation and GH variation for combined values of segment 2 (−R_2_ and −C_2_).

**Figure 3:**
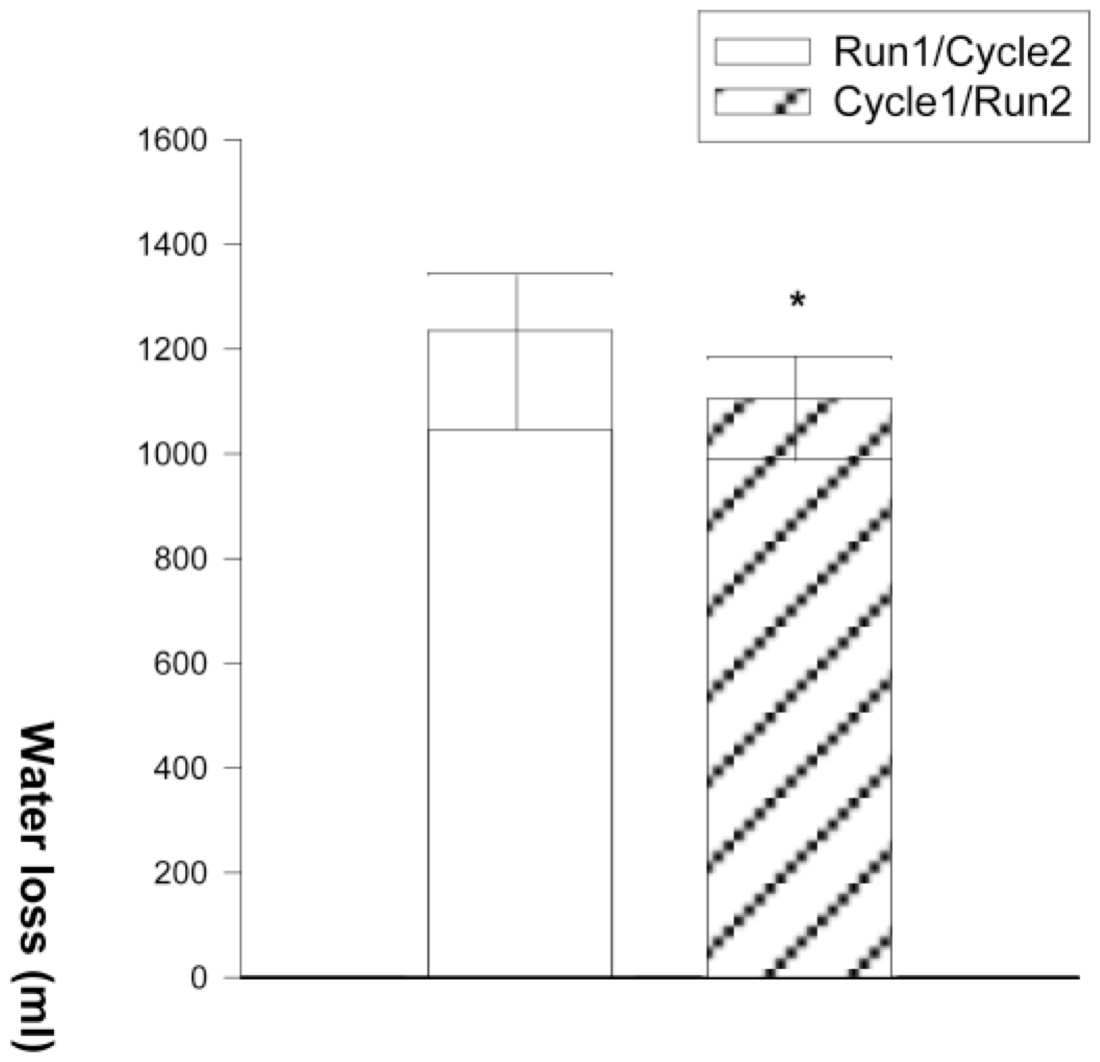
Total water loss. Water loss was measured at the end of each complete trial. Values are represented as mean SD. * *P*<0.05 vs. reversed order trial.

**Figure 4:**
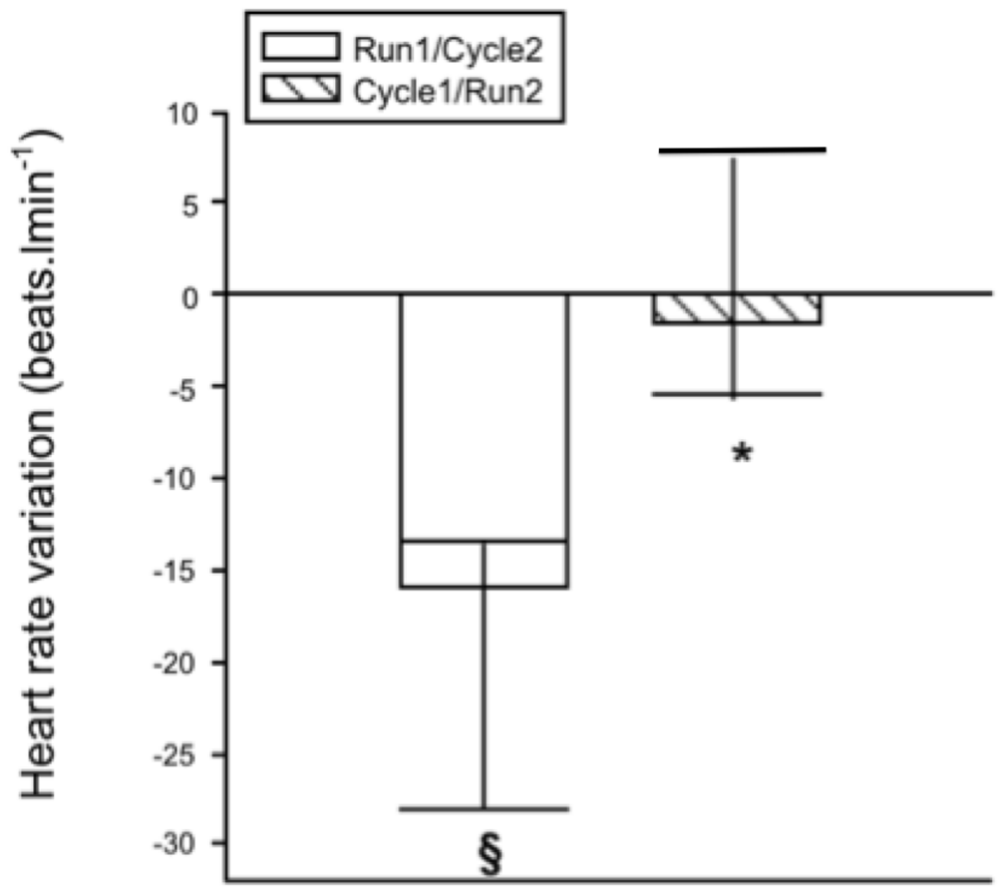
Heart rate variation the during transition. Heart rate variation during the brief active transition of 1 min 30 s was calculated as the difference between the values obtained in the last minute of segment 1 and the value obtained just before the beginning of segment 2. Values are represented as mean SD. *, *P*<0.05 vs. reversed order trial. §, *P*<0.05 vs. 0.

A significant negative correlation was found between the GH-AUC and the plasma volume variation for the combined values obtained in R_2_ and C_2_ (Figure 2). Significant differences were observed between plasma viscosity at rest and in segment 1 and between plasma viscosity at rest and in segment 2 (*P*<0.05). Plasma viscosity observed in C_2_ was not significantly different from that in R_1_, but the value obtained in R_2_ was significantly lower than in C_1_ (*P*<0.05). All values are presented in Table 3. Readers can find elsewhere complementary information on blood parameters observed in similar conditions [13]. Water loss was significantly lower after C_1_-R_2_ than R_1_-C_2_ (1105±163 and 1235±153 ml, respectively; *P*<0.05, Figure 3). The heart rate variation during the transition, i.e., the difference between HR at the last minute of segment 1 and HR immediately before the beginning of segment 2, was calculated. During R_1_-C_2_ this difference was significantly negative (*P*<0.05), while during C_1_-R_2_ this difference was not significant (Figure 4).

## Discussion

The main finding of this study was that the same activities (i.e., cycling and running) performed at the same intensity and duration induced a lower GH increase in R_2_ of a C_1_-R_2_ trial and a plasma volume expansion by reducing sweating.

The GH concentration in segment 1 did not significantly differ between cycling and running. This agrees with a previous study that demonstrated no significant difference in GH response induced by leg cycling and running, when both were performed at the same intensity or percentage of maximal oxygen consumption [25]. Some authors have demonstrated that exercise-induced GH response is determined by both the intensity and duration of exercise [1], [26]. In agreement, our results showed that GH concentration after segment 2 was significantly higher than after segment 1 for all trials. Given that a complete trial was 40 minutes (excluding the 1 min 30 s of transition) of exercise performed at about 75% of maximal oxygen consumption, the finding of higher GH concentrations post-40 minutes–at the end of segment 2–than post-20 minutes–at the end of segment 1–seems logical.

Parameters that increase during exercise should always be interpreted with caution, since most of the apparent changes could be explained by hemoconcentration. For this reason, we corrected GH values for plasma contraction and used these corrected values (GH-AUC) in our interpretation of the results [19], [9]. This led us to observe that the GH increase in R_2_ was significantly lower than that of C_2_. Moreover, the plasma volume variation of R_2_ was significantly different from that of C_2_ and was significantly positive. In accordance with previous studies [9], [27], [28], the plasma viscosity values supported the values for plasma volume variation. The negative plasma variation after each segment 1 was accompanied by significantly higher plasma viscosity in segment 1 than at rest, indicating hemoconcentration as previously observed [13], [15], [16]. After C_2_, no significant plasma volume variation was noted and there was no significant difference in plasma viscosity between R_1_ and C_2_. The plasma viscosity in R_2_ was significantly lower than in C_1_. This corresponds to the fluid shift replacements observed in the subsequent run of cycle-run trials [13], which are accompanied by hemodilution during the subsequent run [13], [14] and which seem to be training-dependent [29]. Thus, the lower GH variation post-R_2_ was concomitant to an expansion of plasma volume. Moreover, a significant negative correlation was found between the plasma volume variation and the GH increase for the combined values of segment 2 (Figure 2). This indicated that, in this segment, the greater the GH increase, the greater the loss in plasma volume. Many studies have suggested that GH acutely stimulates sweating during exercise [2], [3], [4], [5], [6], [7], [8], [9], [10], [30]. Because sweating induces plasma volume filtration in sweat glands and thus plasma volume loss and subsequent hemoconcentration [27], we suggest that the lower GH variation in R_2_ may have induced lower sweating, thereby contributing to volume expansion. This was supported by the water loss measured after the C_1_-R_2_ trial, which was significantly lower than post-R_1_-C_2_. Indeed, although we only globally measured water loss, we suspect that the difference contributed to the expanded plasma volume of the subsequent run. Wells et al. [14] demonstrated expanded plasma volume during the run segment of C_1_-R_2_ trials performed in short-course triathlon conditions. It is interesting to note that we observed the same expansion during the same sequence, even though it was lower. These authors excluded the role of sweating in this phenomenon in the short-triathlon field conditions. However, it should be noted that in their study the triathletes were able to drink ad libitum during the trials. Their results regarding weight loss were thus based on both water intake and loss. In addition, their trials were longer and may have induced a late-occurring mechanism of thermoregulation and hydroregulation different from that observed early on or in shorter trials. In the present study, we suggest that sweating was implicated in the expanded plasma volume observed in the sequential running performed at the same duration and intensity as the preceding cycling and the segments performed in reversed order. Furthermore, in a previous study we hypothesized that because GH may stimulate water loss by activating sweating, exercise-induced GH response may in turn be under the control of baroreceptor activity [9].

We noted heart rate variation during the brief active transition of each trial. During R_1_-C_2_, this variation was significantly negative (Figure 4) because of the well-documented rapid restoration of vagal control generated by the rapid ending of intense exercise, which has been shown to be increased in athletes [31], [32], [33]. During the C_1_-R_2_ trial, however, the variation was not statistically significant. We thus assumed that the normally rapid decrease in heart rate generated by the termination of exercise was compensated by an increase in heart rate induced by the postural change-activated baroreceptor reflex [11], [12]. This would explain the absence of a significant fall in heart rate during the C_1_-R_2_ transition. Thus, in accordance with a previous study [9], we hypothesize that if this baroreceptor activation truly occurred during the active transition, it would initiate the inhibition of GH secretion. Because the active transition was very short (only 1 min 30 s), the inhibitory effect initiated during this short period may have led to the reduced GH increase observed in R_2_.

From a methodological point of view, it should be noted that we took only one blood sample at the end of segment 1, which we then considered as the reference time for both the end of segment 1 and just before the beginning of segment 2. Similarly, we did not measure water loss after each segment, but only at the end of a complete trial. We did this because the active transition period was very brief–only 1 min 30 s–and we wanted to ensure minimal disturbance of the trial.

## Conclusions

This study showed that the lower GH variation in R_2_ of a C_1_-R_2_ trial performed at the same relative intensity and for the same duration contributed to the plasma volume expansion by reducing sweating, suggested role of GH in the sweat process.

Furthermore, we hypothesize that this lower GH variation was induced by a baroreceptor reflex [9], activated by the specific postural change from cycling to running during the brief active transition. This phenomenon observed during R_2_ needs to be considered beyond the transition phase. While triathletes would be more dehydrated in C_2_ vs R_2_, we recommend to pay particular attention to their hydration status during multi cycle-run and run-cycle training sessions as well as during competitions, in drinking systematically during R_1_ which could limit a potential dehydration during C_2_. However, this does not exclude the interest and the importance to hydrate during C_1_, which is more confortable than during running, and gives the opportunity to freely hydrate.
